# To block or not to block: The adaptive manipulation of plague transmission

**DOI:** 10.1002/evl3.111

**Published:** 2019-03-27

**Authors:** Sylvain Gandon, Louise Heitzmann, Florent Sebbane

**Affiliations:** ^1^ CEFE UMR 5175 CNRS ‐ Université de Montpellier ‐ Université Paul‐Valéry Montpellier – EPHE 1919 route de Mende 34293 Montpellier France; ^2^ Inserm, Univ. of Lille, CNRS, CHU Lille, Institut Pasteur de Lille, U1019—UMR8204—CIIL—Center for Infection and Immunity of Lille F‐59000 Lille France

**Keywords:** Biofilm, epidemiology, evolution, flea blockage, multi‐host pathogens, parasite manipulation, plague, transmission

## Abstract

The ability of the agent of plague, *Yersinia pestis*, to form a biofilm blocking the gut of the flea has been considered to be a key evolutionary step in maintaining flea‐borne transmission. However, blockage decreases dramatically the life expectancy of fleas, challenging the adaptive nature of blockage. Here, we develop an epidemiological model of plague that accounts for its different transmission routes, as well as the within‐host competition taking place between bacteria within the flea vector. We use this theoretical framework to identify the environmental conditions promoting the evolution of blockage. We also show that blockage is favored at the onset of an epidemic, and that the frequencies of bacterial strains exhibiting different strategies of blockage can fluctuate in seasonal environments. This analysis quantifies the contribution of different transmission routes in plague and makes testable predictions on the adaptive nature of blockage.

**Impact Summary**

Plague transmission relies on the ability of infected fleas to inoculate *Yersinia pestis* bacteria to its vertebrate hosts. The production of a biofilm by the bacteria blocks the foregut of the flea and increases infectivity. However, the adaptive nature of blockage remains controversial because it has a massive survival cost on the infected fleas and reduces dramatically the length of the infection: an extreme form of the classical virulence‐transmission tradeoff. Here, we develop a comprehensive model of the multiple routes of plague transmission, we determine when blockage can be considered as an adaptive manipulation of its flea vector, and we generate several testable predictions on the evolution of plague in both endemic and epidemic situations.

*Yersinia pestis* is the bacterium that caused three plague pandemics and had a profound effect on human history (Bramanti et al. 2016). A combination of comparative genomic analyses and experimental studies has unveiled the different evolutionary steps leading to the emergence and the spread of this deadly pathogen for numerous mammals, including humans. *Y. pestis* recently emerged from *Yersinia pseudotuberculosis*, a food‐ and waterborne enteric pathogen causing a benign disease of the digestive tract in humans (Achtman et al. 1999; Sun et al. 2014; Hinnebusch et al. 2016; Valtueña et al. 2017). Only a handful of genetic events, including acquisition of genes by horizontal transfer and loss of functional genes, led to the production of flea‐borne transmission of plague (Chouikha and Hinnebusch 2012; Sun et al. 2014; Hinnebusch et al. 2016; Hinnebusch et al. 2017). Notably, the horizontal acquisition of the *Yersinia* murin toxin gene (*ymt*) that protects from a bacteriolytic agent generated during the digestion of the blood meal has been essential to colonize the flea's midgut and foregut (Hinnebusch et al. 2002). Loss of a functional urease accessory protein UreD due to the insertion of a single nucleotide in the *ureD* locus reduced the toxicity of the ancestral strain, thereby prolonging the duration of infection in the vector (Chouikha and Hinnebusch 2014; Sebbane et al. 2001). Lastly, a series of other pseudogenizations (i.e., genetic mutations that lead to gene inactivation) led to the loss of the functional accessory regulatory protein RcsA and of two phospodisterases (PDE) that unlocked the pre‐existing capability of the ancestral strain to form a biofilm, thanks to the *hmsHFRS* operon (Hinnebusch et al. 1996, 2017, Sun et al. 2008, 2014). The formation of a biofilm enabled the persistent colonization of the proventriculus and, ultimately, the blockage of flea's gut (Hinnebusch et al. 1996, 2017).

When the proventriculus of the flea is blocked, the biofilm prevents the incoming blood from entering the midgut. The blood meal is contaminated upon contact with the bacterial mass, and is regurgitated at the flea‐bite site, leading to transmission of plague (Bacot and Martin 1914; Hinnebusch et al. 1996). Another consequence of the blockage is an increase in the biting rate as the flea starves to death. Therefore, blockage is often viewed as a key adaptation of *Y. pestis* because it boosts bacterial transmission by increasing both infectivity (the number of bacteria inoculated in a new host) and the biting rate of infected fleas (Hinnebusch et al. 1996, 2017). Yet, the adaptive nature of blockage is challenged by the fact that it drastically increases the mortality rate of the flea (Hinnebusch et al. 1996, 2017). Besides, a combination of experimental observations and empirical studies suggest that other routes of transmission may be involved in plague epidemics (Mollaret 1963; Eisen et al. 2006; Webb et al. 2006; Eisen et al. 2007; Eisen et al. 2008; Chouikha and Hinnebusch 2012; Eisen et al. 2015; Hinnebusch et al. 2016). In particular, some flea transmission may also occur in an early phase of the infection of unblocked flea (Eisen et al. 2006; Webb et al. 2006; Eisen et al. 2007; Eisen et al. 2015). In other words, blockage may be viewed as a by‐product of the colonization of the foregut but not as an adaptive manipulation of the biting rate of its insect vector.

BOX: The ecology of plague transmission 1 Plague is caused by the Gram‐negative bacterium *Y. pestis* and is mainly a disease of rodents and their associated fleas. *Y. pestis* can also infect a large diversity of mammals, including humans, but most of these infections are considered accidental. Plague remains endemic in many parts of the world (Africa, Asia, America, and South‐Eastern Europe) and it occurs in a variety of ecosystems (arid, semi‐arid, steppe, tropical mountainous) where climatic conditions are favorable for the development of competent rodent and flea species. In endemic populations, the disease is circulating in rodent populations composed of individuals with variable resistance to the disease (enzootic cycle). But the introduction of plague in highly susceptible populations can lead to explosive spread and massive mortality (epizootic cycle). These epidemics may be driven by climatic factors and by the fluctuations in the density of suitable hosts.

*Y. pestis* can infect the mammal through inhalation, ingestion, or direct contact with a wound, but the main route of transmission results from the bite of an infected flea. There are more than 2500 species of fleas, but only a small number of them have been reported as naturally infected and an even smaller number have been considered as active vectors of plague transmission. All naturally infected flea species appear to have the potential to effectively transmit the disease within the first 5 days after infection (early transmission) while only some species can transmit the disease for one month after the contaminated meal. This late transmission is related to the ability of *Y. pestis* to produce a biofilm and to block the foregut of the flea (figure). Blockage of the flea modifies the biting behavior of the flea and increases bacterial transmission. But blockage starves the flea and often results in flea death after a few days, unless the biofilm is broken by the uptake of a new blood meal and results in reversion to the unblocked stage. For excellent reviews on plague transmission, see Gage and Kosoy 2005, Gage and Kosoy 2006, and Hinnebusch et al. 2017.

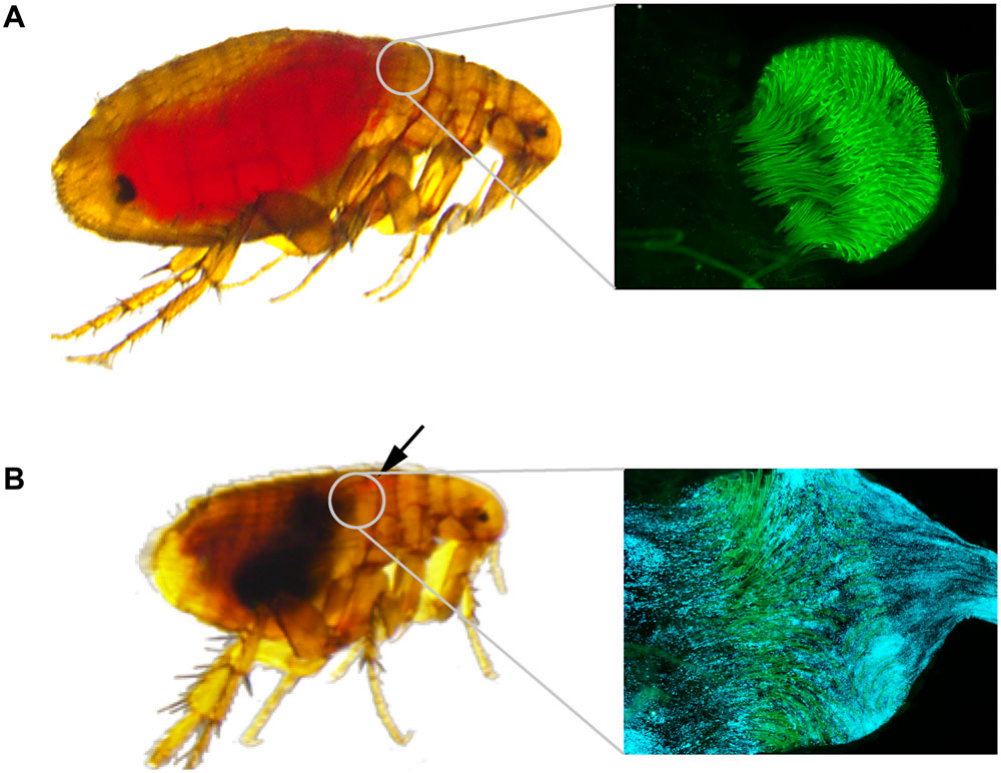

**Figure**. *Y. pestis* blocks the flea foregut. (A) Upon ingestion of a blood meal, healthy fleas contain a fresh meal in their midgut. (B) Six days after ingestion of an infected blood meal, infected fleas display blockage of their proventriculus. Blocked fleas are smaller than healthy fleas and, after an attempt to feed, display fresh red blood in the proventriculus and/or the esophagus (black arrow) but not in the midgut. Blockage results from the formation of biofilm by *Y. pestis* within the flea's proventriculus. This is illustrated by the fluorescence microscopy images showing the absence (A) and presence (B) of numerous *Y. pestis* bacteria expressing a fluorescent protein (in blue) located within the proventriculus's pines (with green autofluorescence) and the esophagus.

The biology of plague is complex and involves multiple routes of transmission via a diversity of host and vector species (see Box 1 for a brief summary on the ecology of plague transmission). The present study is an attempt to quantify the epidemiological and evolutionary implications of the blockage of fleas for the dynamics of *Y. pestis*. First, to evaluate the relative importance of blockage on plague transmission, we develop a theoretical framework that accounts for the multiple routes of transmission of *Y. pestis*. We use this framework to derive the basic reproduction number of the pathogen. In a second step, we expand this theoretical framework to study the evolution of the propensity to block the flea. To analyze pathogen evolution, we study the competition between bacterial strains with varying blockage strategies. This competition takes place at a between‐host level when bacteria are trying to infect new hosts. But bacteria may also compete within‐host when, for instance, a flea is coinfected by different strains after feeding on two infected hosts. We derive threshold conditions allowing the invasion of a mutant strain with a specific blockage strategy in a stable environment. These threshold conditions, however, are derived under the assumption that the pathogen has reached an endemic equilibrium (see Box 1). In a third and final step, we relax this equilibrium assumption because the dynamics of plague is often characterized by sudden epidemics and major fluctuations in incidence through time. We analyze the evolution of plague during epidemics and show how bacteria with different rates of blockage can fluctuate in a seasonal environment. In particular, we predict that the rate of blockage is expected to rise at the onset of epidemics even when blockage is selected against in the long term. All these theoretical predictions are discussed in the light of available data obtained on *Y. pestis* in laboratory controlled experiments.

## The Model

**Figure 1:**
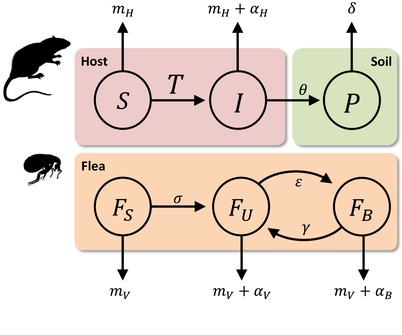
**Our epidemiological model accounts for multiple routes of plague transmission**. The pathogen circulates among three different habitats: (1) a vertebrate host, (2) a flea vector, and (3) the soil (see description of life cycle in the main text and the definition of all parameters in Table S1). The rate at which uninfected hosts become infected is determined by the sum of the force of infection from the different compartments of this system: $T=\beta_{H}I+\beta_{P}P+\sigma \beta_{U}F_{U}+\sigma \beta_{B}F_{B}$.

Our epidemiological model is an attempt to capture the main components of the complex life cycle of *Y. pestis* described in Box 1. In particular, our model accounts for the fact that *Y. pestis* bacteria can live and/or persist in three different habitats: (i) a vertebrate host (usually a rodent), (ii) a flea, and (iii) the soil (see Fig. 1 for a schematic description of the model through these three different compartments). We assume that the vertebrate host has an intrinsic reproduction rate $\lambda_{H}$ and a natural mortality rate $m_{H}$. The flea vector is assumed to have an intrinsic reproduction rate $\lambda_{F}$ and a natural mortality rate $m_{F}$. For the sake of simplicity, we assume these growth rates to be constant but assuming density‐dependent growth rates do not affect qualitatively the results we present below (see Materials and Methods section). Because we are interested in plague evolution, we assume that multiple bacterial strains can circulate. We note that $P_{i}$ is the density of the free‐living stage of the strain *i* (we assume that these propagules can persist in the environment but cannot replicate) and $I_{i}$ is the density of hosts infected with the strain *i*. The parameter σ measures the biting rate of fleas on the host. After feeding on a host infected with strain *i*, the infected flea is assumed to be “unblocked” (state $F_{U,i}$). Infectious fleas can become “blocked” (state $F_{B,i}$) and the transition between the “unblocked” and the “blocked” states occurs at a rate $\varepsilon_{i}$ (the rate of blockage), which is assumed to vary among different strains of *Y. pestis*. We also assume that blocked fleas can become unblocked (return to the state $F_{U,i}$, as observed in Bacot and Martin 1914) at a constant rate γ. Infection increases the mortality of the host ($\alpha_{H}$), and the mortality of both the blocked and the unblocked fleas ($\alpha_{B}$ and $\alpha_{U}$, respectively). It is important to note that blockage has a major impact on flea survival ($\alpha_{B}>\alpha_{U}$) (Hinnebusch et al. 1996, 2017). Hence, bacterial strains that promote blockage are associated with higher virulence in the flea because blockage decreases survival. The host can acquire the infection horizontally from other infected hosts at a rate $\beta_{H}I_{i}$, from the propagules in a contaminated environment at a rate $\beta_{P}P_{i}$ and from the infected vectors at rates $\sigma \;\beta_{U}F_{U,i}$ and $\sigma \;\beta_{B}F_{B,i}$. The parameters $\beta_{H}$, $\beta_{P}$, $\beta_{U}$, and $\beta_{B}$ modulate the relative importance of these four different routes of transmission. Crucially, experimental studies have demonstrated that blockage increases the infectiousness of fleas and thus $\beta_{B}>\beta_{U}$ (Hinnebusch et al. 1996, 2017; Lorange et al. 2005; Sebbane et al. 2009). The density of the total host population is denoted as $N_{H}=S+I$. Similarly, the density of the total flea population is denoted as $N_{F}=F_{S}+F_{U}+F_{B}$. This life cycle can be summarized in the following system of differential equations (see Table S1 for the definition of all the parameter of this model):

(1) $$\begin{array}{ccc} \dot{S} & = & \lambda_{H}-\left(\beta_{H}I_{i}+\beta_{P}P_{i}+\sigma \beta_{U}F_{U,i}+\sigma \beta_{B}F_{B,i}+m_{H}\right)S\\ {\dot{F}}_{S} & = & \lambda_{F}-\left(\sigma \sum_{i}I_{i}+m_{F}\right)F_{S}\\ {\dot{I}}_{i} & = & \left(\beta_{H}I_{i}+\beta_{P}P_{i}+\sigma \beta_{U}F_{U,i}+\sigma \beta_{B}F_{B,i}\right)S-\left(m_{H}+\alpha_{H}\right)I_{i}\\ {\dot{F}}_{U,i} & = & \sigma F_{S}I_{i}+\gamma F_{B,i}-\left(m_{F}+\alpha_{U}+\varepsilon_{i}\right)F_{U,i}+\sigma \sum_{j\neq i}s\left[\varepsilon_{j},\varepsilon_{i}\right]\\ & & \times \;I_{i}F_{U,j}-\sigma \sum_{j\neq i}s\left[\varepsilon_{i},\varepsilon_{j}\right]I_{j}F_{U,i}\\ {\dot{F}}_{B,i} & = & \varepsilon_{i}F_{U,i}-\left(m_{F}+\alpha_{B}+\gamma \right)F_{B,i}\\ {\dot{P}}_{i} & = & \theta I_{i}-\delta P_{i}. \end{array}$$

The above model accounts also for the competition taking place between bacterial strains in the early stage of the infection (i.e., in unblocked fleas). Indeed, when an unblocked flea infected with strain *i* feeds on a host infected by strain *j*, the superinfection function $s[\varepsilon_{i},\varepsilon_{j}]$ determines the probability that strain *i* is replaced by strain *j*. We assume that the competitivity of the bacteria may be associated with the propensity to form biofilms and to block the flea. We used the following function to model superinfection:

(2) $$s\left[\varepsilon_{i},\varepsilon_{j}\right]=\frac{s_{0}}{s_{0}+\left(1-s_{0}\right)e^{\frac{-{s_{0}}^{\prime }\left(\varepsilon_{j}-\varepsilon_{i}\right)}{s_{0}\left(1-s_{0}\right)}}},$$

where $s_{0}=s\left[\varepsilon_{i},\varepsilon_{i}\right]$ is the value of the probability of superinfection at the origin (when both strains have the same value of ε) and $s_{0}^{\prime }=ds\left[\varepsilon_{i},\varepsilon_{j}\right]/d\varepsilon_{j}{|}_{\varepsilon_{i}=\varepsilon_{j}}$ is the slope of the superinfection function at the origin (Fig. S1).

Note that we neglect the possibility that competition may occur in blocked fleas and in vertebrate hosts because the bacterial density reached in blocked fleas and in infected hosts hampers invasion by new strains. This is arguably a very simplified view of the way within‐host competition among bacterial strains may occur in this system. Yet, as we will see below, the simplicity of this model shows the potential implications of within‐host competition on plague evolution and leads to novel adaptive hypothesis for the evolution of blockage.

## Epidemiology and Evolution in a Stable Environment

First, we focus on a scenario where the population of the bacteria is monomorphic and all the parameters of the model are constant. The basic reproduction ratio *R*_0_ of the pathogen is given by (see Materials and Methods section):

(3) $$\begin{array}{ccc} R_{0} & = & \frac{N_{H}}{m_{H}+\alpha_{H}}\left(\beta_{H}+\beta_{P}\frac{\theta }{\delta }\right.\\ & & \left.+\;\beta_{U}\frac{\sigma^{2}N_{F}\left(m_{F}+\gamma +\alpha_{B}\right)}{A}+\beta_{B}\frac{\sigma^{2}\varepsilon N_{F}}{A}\right) \end{array}$$

with $A=m_{F}\left(m_{F}+\gamma +\varepsilon \right)+\alpha_{U}\left(m_{F}+\gamma \right)+\alpha_{B}\left(m_{F}+\alpha_{U}+\varepsilon \right)$ and where $N_{H}$ and $N_{F}$ are derived at the disease free equilibrium: $\left(S,I\right)=\left(\frac{\lambda_{H}}{m_{H}},0\right)$ and $\left(F_{S},F_{U},F_{B}\right)=\left(\frac{\lambda_{F}}{m_{F}},0,0\right)$. The above expression is useful to identify the relative importance of the different routes of transmission on the epidemiology of plague. Indeed, each term in the parenthesis is associated with the contribution of each of the four different routes of transmission to *R*_0_: (i) direct horizontal transmission by contact between uninfected and infected hosts, (ii) transmission via propagules in the environment, (iii) transmission via unblocked fleas, and (iv) transmission via blocked fleas.

This expression is also particularly useful to identify the conditions promoting the ability of the pathogen to trigger an epidemic in an uninfected host population. When $R_{0}>1$, the pathogen can invade the host population and the system ultimately reaches an endemic equilibrium in which the pathogen persists in the different compartments (the notation $\bar{X}$ is used to refer to the equilibrium density of the variable *X* at this endemic equilibrium). Numerical exploration of the system (1) revealed that this endemic equilibrium was always locally stable.

In the following, we study the long‐term evolutionary dynamics of plague using the classical formalism of Adaptive Dynamics, in which mutation rate is assumed to be low, that allows decoupling evolutionary and epidemiological dynamics (Metz et al. 1992; Geritz et al. 1998; Waxman and Gavrilets 2005; Kisdi and Geritz 2010). To study plague evolution, we derive the invasion fitness per‐generation of a “mutant” strain that has the strategy $\varepsilon_{m}$, at the endemic equilibrium set by a resident population of the pathogen which has the strategy ε (Hurford et al. 2010) (see Materials and Methods section):

(4) Rm=S¯HmH+αHβH+βPθδ+σ2AmβUmF+γ+αB+βBεmF¯S+sε,εmF¯Uθδ

with $A_{m}=m_{F}\left(m_{F}+\gamma +\varepsilon_{m}\right)+\alpha_{U}\left(m_{F}+\gamma \right)+\alpha_{B}\left(m_{F}+\alpha_{U}+\varepsilon_{m}\right)+\sigma s\left[\varepsilon_{m},\varepsilon \right]\bar{I}\left(m_{F}+\gamma +\alpha_{B}\right)$. The mutant will invade the resident population if $R_{m}>1$ and this invasion fitness can be used to derive the gradient of selection on blockage at the endemic equilibrium (i.e., ${\bar{S}}_{H}$, ${\bar{F}}_{S}$, ${\bar{F}}_{U}$, and $\bar{I}$) set by the resident strategy.

We used this invasion fitness to identify the conditions leading to the evolution of higher rates of blockage (see Materials and Methods section). In particular, under the assumption that the superinfection function is constant and equal to *s*_0_, we find that higher rates of blockage are selected for when:

(5) $$\frac{\beta_{B}}{m_{F}+\alpha_{B}}>\frac{\beta_{U}}{m_{F}+\alpha_{U}+\sigma s_{0}\bar{I}}.$$

Hence, in spite of the complexity of the life cycle, the evolution of blockage boils down to a very simple condition that does not depend on the other routes of transmission. The left and the right hand sides of equation (5) measure of the relative “quality” of blocked and unblocked fleas, respectively. The quality of a vector depends on the instantaneous rate of transmission ($\beta_{B}$ and $\beta_{U}$) but also the duration of the infection that is modulated by the mortality rates ($m_{F}$, $\alpha_{U}$, and $\alpha_{B}$) as well as the rate of superinfection in unblocked fleas ($\sigma s_{0}\bar{I}$). When condition (5) is satisfied, the blocked fleas are better vectors than unblocked fleas and blockage evolves to maximal values. In contrast, when condition (5) is not satisfied, unblocked fleas are better vectors, blockage does not evolve, and the evolutionary stable strategy is $\varepsilon^{*}=0$.

The invasion condition can also be used to determine the conditions favoring the evolution of blockage when the probability of superinfection depends on the investment in blockage of the competing strains (i.e., ${s_{0}}^{\prime }\neq 0$). For instance, under the simplifying assumption that the resident strain does not block (ε=0) the condition for the invasion of a mutant strain that blocks the flea is:

(6) $$\frac{\beta_{B}}{m_{F}+\alpha_{B}}>\frac{\beta_{U}}{m_{F}+\alpha_{U}+\sigma s_{0}\bar{I}}-{s_{0}}^{\prime }B,$$

where $B=\frac{\beta_{U}\left(m_{F}+\alpha_{B}+\gamma \right)}{m_{F}+\alpha_{B}}\left(\frac{{\bar{F}}_{U}}{{\bar{F}}_{S}+s_{0}{\bar{F}}_{U}}+\frac{\sigma \bar{I}}{m_{F}+\alpha_{U}+\sigma s_{0}\bar{I}}\right)$.

**Figure 2:**
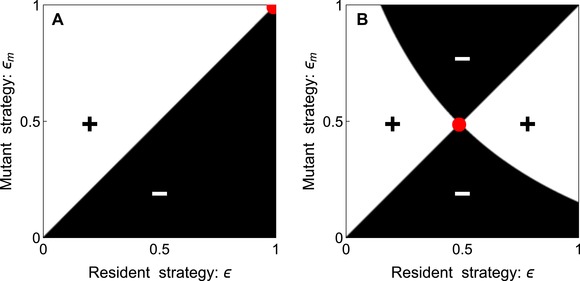
**Pairwise invasibility plot on the rate of blockage**. We use equation (4) to plot the ability of the mutant strategy $\varepsilon_{m}$ to invade a resident population with strategy ε. When $R_{m}>1$ the mutant can invade (white) and when $R_{m}<1$ the mutant fails to invade the resident population (black). In (A) $s_{0}^{\prime }=0$ and in (B) $s_{0}^{\prime }=-0.4.$ Pairwise invisibility plots can be used to find the ultimate evolutionary outcome (red dot) but also to identify pairs of strategies that can coexist. Panel (B) shows that an intermediate strategy can be evolutionary stable. Other parameter values: $\lambda_{H}=0.01,\lambda_{F}=0.1,\;\gamma =0.2,\;\theta =0.5,\;\sigma =0.25,\;\delta =1,\;m_{H}=0.004,\;m_{F}=0.02,\;\alpha_{H}=0.01,\alpha_{U}=0.02,\;\alpha_{B}=0.2,\;\beta_{H}=0.001,\;\beta_{P}=0.01,\;\beta_{U}=0.005,\;\beta_{B}=0.025,\;s_{0}=0.5$.

The above condition shows that if the ability to block the flea is associated with a higher competitive ability of the bacteria (i.e., $s_{0}^{\prime }>0$), blockage can evolve more readily. In contrast, if the production of a biofilm is costly and induces a lower competitive ability (i.e., $s_{0}^{\prime }<0$), it is more difficult to evolve blockage. Adding such a cost on biofilm production allows some intermediate blockage strategy to be evolutionary stable (Fig. 2).

## Evolution in a Fluctuating Environment

**Figure 3:**
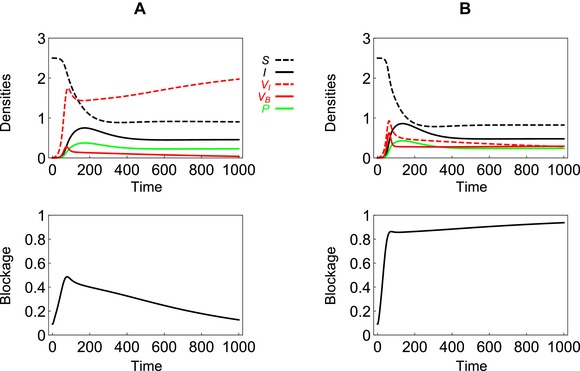
**Epidemiology and evolution of plague during an epidemic**. We present the epidemiological dynamics and the evolutionary dynamics in the absence of superinfection. The top figures show the dynamics of the densities of the different compartments of the model during an epidemic. The bottom figures show the dynamics of the mean value of the blockage strategy. We allow competition between two strains with different blockage strategies (i.e., ε=0 or 1). In panel (A), $\beta_{B}=0.025$ and blockage is maladaptive according to condition (5) in the absence of superinfection (i.e., $\frac{\beta_{B}}{m_{F}+\alpha_{B}}\approx 0.09<\frac{\beta_{U}}{m_{F}+\alpha_{U}}=0.125$) but blockage is selected for at the beginning of the epidemic. In panel (B), $\beta_{B}=0.04$ and blockage is adaptive according to condition (5) in the absence of superinfection (i.e.,$\frac{\beta_{B}}{m_{F}+\alpha_{B}}\approx 0.15>\frac{\beta_{U}}{m_{F}+\alpha_{U}}=0.125$). Other parameter values (see **Materials and Methods section** for more details about the simulation procedure): $\lambda_{H}=0.01,\;\lambda_{F}=0.1,\;\gamma =0.2,\;\theta =0.5,\;\sigma =0.4,\;\delta =1,\;\;m_{H}=0.004,\;m_{F}=0.02,\;\alpha_{H}=0.01,\;\alpha_{U}=0.02,\;\alpha_{B}=0.25,\;\beta_{H}=0.001,\;\beta_{P}=0.01,\;\beta_{U}=0.005,\;\beta_{B}=0.025,\;s_{0}=0.5$.

Because plague dynamics is often characterized by dramatic temporal fluctuations (Schmid et al. 2015; Stenseth et al. 2008), we examined the evolution of blockage away from the endemic equilibrium. Numerical simulations show that at the onset of an epidemic, a mutant strain with a higher ability to block the flea can increase in frequency (Fig. 3) even if this blockage strategy does not verify conditions (5) or (6). To understand pathogen evolution during this transient phase of the epidemics, it is important to track both the “frequency” of the different strains and the “densities” of the pathogen in the different compartments of the model (Day and Gandon 2006; Day and Gandon 2007; Berngruber et al. 2013; Lélu et al. 2013). In the following, we derive the dynamics of the frequencies $p_{i}^{X}$, of the strain *i* in the compartment *X*:

(7) $$\begin{array}{ccc} {\dot{p}}_{i}^{I} & = & \left(\beta_{P}\frac{P}{I}\left(p_{i}^{P}-p_{i}^{I}\right)+\sigma \beta_{U}\frac{F_{U}}{I}\left(p_{i}^{F_{U}}-p_{i}^{I}\right)\right.\\ & & \left.+\;\sigma \beta_{B}\frac{F_{B}}{I}\left(p_{i}^{F_{B}}-p_{i}^{I}\right)\right)S\\ {\dot{p}}_{i}^{F_{U}} & = & \sigma \frac{I}{F_{U}}F_{S}\left(p_{i}^{I}-p_{i}^{F_{U}}\right)+\gamma \frac{F_{B}}{F_{U}}\left(p_{i}^{F_{B}}-p_{i}^{F_{U}}\right)-\left(\varepsilon_{i}-{\bar{\varepsilon }}^{F_{U}}\right)p_{i}^{F_{U}}\\ & & +\;\sigma I\left(\sum_{j\neq i}s\left[\varepsilon_{j},\varepsilon_{i}\right]p_{i}^{I}p_{j}^{F_{U}}-\sum_{j\neq i}s\left[\varepsilon_{i},\varepsilon_{j}\right]p_{j}^{I}p_{i}^{F_{U}}\right)\\ {\dot{p}}_{i}^{F_{B}} & = & \frac{F_{U}}{F_{B}}\left(\left(\varepsilon_{i}-{\bar{\varepsilon }}^{F_{U}}\right)p_{i}^{F_{U}}-{\bar{\varepsilon }}^{F_{U}}\left(p_{i}^{F_{B}}-p_{i}^{F_{U}}\right)\right)\\ {\dot{p}}_{i}^{P} & = & \frac{\theta I}{P}\left(p_{i}^{I}-p_{i}^{P}\right) \end{array}$$

where ${\bar{\varepsilon }}^{F_{U}}=\sum_{i}p_{i}^{F_{U}}\varepsilon_{i}$ is the average value of blockage in unblocked fleas.

Focusing on the dynamics of mutant frequency is particularly useful to understand the interplay between epidemiology and evolution. For instance, let us focus on the scenario in which two bacterial strains compete: a mutant strain that blocks the fleas at a rate $\varepsilon_{m}$ and a resident strain that never blocks the fleas. In this case only the mutant can block the fleas and thus, $p_{m}^{F_{B}}=1$. If we neglect superinfections and assume the initial frequency of the mutant is low, the above dynamical system reduces to:

(8) $$\begin{array}{ccc} {\dot{p}}_{i}^{I} & = & \left(\beta_{P}\frac{P}{I}\left(p_{m}^{P}-p_{m}^{I}\right)+\sigma \beta_{U}\frac{F_{U}}{I}\left(p_{m}^{F_{U}}-p_{m}^{I}\right)+\sigma \beta_{B}\frac{F_{B}}{I}\left(1-p_{m}^{I}\right)\right)S\\ {\dot{p}}_{i}^{F_{U}} & = & \sigma \frac{I}{F_{U}}F_{S}\left(p_{m}^{I}-p_{m}^{F_{U}}\right)+\gamma \frac{F_{B}}{F_{U}}\left(1-p_{m}^{F_{U}}\right)-\varepsilon_{m}p_{m}^{F_{U}}\\ {\dot{p}}_{i}^{P} & = & \frac{\theta I}{P}\left(p_{m}^{I}-p_{m}^{P}\right). \end{array}$$

Initially, the mutant frequency is expected to be low in all the other three compartments of the model (*I*, $F_{U}$, and *P*) that yields the following approximation for the change in mutant frequency in the infected host compartment: ${\dot{p}}_{i}^{I}\approx \sigma \beta_{B}\frac{F_{B}}{I}S$. This indicates that the frequency of a mutant strain that blocks the fleas is initially increasing in the infected host compartment. This initial increase occurs even if the mutant is ultimately selected against (Fig. 3). This transient selection for the mutant is due to the fitness benefit associated with higher transmission rates when there are a lot of susceptible hosts around (Berngruber et al. 2013; Lélu et al. 2013).

**Figure 4:**
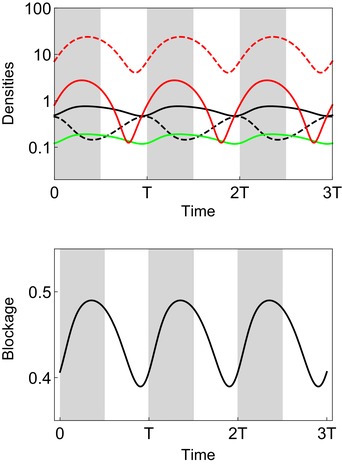
**Epidemiology and evolution of plague in a seasonal environment**. We allow the growth rate of the flea population $\lambda_{F}\left(t\right)=1+Sin\left(2\pi t/T\right)$ to vary periodically (T=200) because of seasonality (the shaded area indicates time when the growth rate is above average, that is, when $\lambda_{F}\left(t\right)>1$). We assume that two bacterial strains are competing but we do not allow for superinfection: one strain never blocks the flea ($\varepsilon_{1}=0$) and another strain can block infected fleas ($\varepsilon_{2}=1$). The two strains coexist, although blockage is maladaptive according to condition (5) in the absence of superinfection (i.e., $\frac{\beta_{B}}{m_{F}+\alpha_{B}}\approx 0.119<\frac{\beta_{U}}{m_{F}+\alpha_{U}}=0.125$) but their relative frequency fluctuates in synchrony with the incidence of the disease in the vector. Other parameter values (see **Materials and Methods section** for more details about the simulation procedure): $\lambda_{H}=0.01,\;\gamma =0.2,\;\theta =0.5,\;\sigma =0.3,\;\delta =2,\;\;m_{H}=0.004,\;m_{F}=0.02,\;\alpha_{H}=0.01,\;\alpha_{U}=0.02,\;\alpha_{B}=0.3,\;\beta_{H}=0.001,\;\beta_{P}=0.01,\;\beta_{U}=0.005,\;\beta_{B}=0.038$.

The analysis of transient evolution of blockage is also useful to understand the influence of seasonal variations of the environment. Fluctuations in temperature and humidity are likely to impose periodic variations in the densities of multiple hosts and vectors of plague (Moore et al. 2015; Ngeleja et al. 2018). These fluctuations drive periodic fluctuations of the incidence of the infection, maintaining the pathogen away from the endemic equilibrium (epizootic cycles, see Box 1). We explored the influence of a periodic fluctuation in the growth rate of the flea population on the evolution of blockage (Fig. 4). We show that seasonality favors different blockage strategies in different phases of these recurrent epidemics. As discussed above, blockage is selected for at the onset of the epidemics, and it is selected against when the epidemic is fading away. This fluctuating selective pressure on blockage allows the long‐term coexistence of bacterial strains with different rates of biofilm production (Fig. 4).

## Discussion

The emergence and the evolution of plague results from a series of adaptations that increased the efficacy of flea‐borne transmission of *Y. pestis* (Sun et al. 2014; Hinnebusch et al. 2016; Hinnebusch et al. 2017). But whether the blockage of the flea is an adaptation remains a controversial issue (Eisen et al. 2006; Eisen et al. 2007; Eisen et al. 2015; Hinnebusch et al. 2017). Our analysis is an attempt to clarify the conditions that can promote or hamper the evolution of blockage. Here, we consider a situation in which a bacterial mutant with a distinct blockage strategy is introduced in a population of *Y. pestis* and we determine if such a mutant can invade or not. For instance, different genetic variants in the *hmsHFRS* operon are known to affect dramatically the colonization of the proventriculus and the formation of a biofilm: The *hmsFRS+* mutant is known to yield flea blockage whereas *hmsFRS‐* never blocks the fleas and the mortality of fleas blocked by the *hmsFRS+* mutant is considerably larger than unblocked fleas (Hinnebusch et al. 1996; Hinnebusch et al. 2017). Does the gain in transmission due to blockage compensate this increased mortality? Our analysis is an attempt to answer this question. More specifically, the condition (5) shows that blockage is adaptive, in the absence of within‐flea competition, if the ratio of mortality rates between blocked and unblocked fleas is lower than the ratio of transmission rates between blocked and unblocked fleas:

(9) $$\frac{m_{F}+\alpha_{B}}{m_{F}+\alpha_{U}}<\frac{\beta_{B}}{\beta_{U}}.$$

Available data on blocked and unblocked rat flea *Xenopsylla cheopsis* (one of the main flea vector) suggests that that the life expectancy of a blocked flea is around 2 days while the life expectancy of an infected (but unblocked) flea is around 100 days (Hinnebusch et al. 1996; Lorange et al. 2005; Hinnebusch et al. 2017; Hinnebusch et al. 2017). The ratio between mortality rates of blocked and unblocked fleas is thus expected to be around 50. In other words, condition (9) indicates that transmission rate of blocked fleas must be 50 times higher than transmission rate or unblocked fleas for blockage to be adaptive. Available experimental data on *X. cheopsis* suggests that transmission of blocked fleas is likely to be much higher than this threshold value. First, the ratio of the biting rates of blocked and unblocked fleas is likely to be higher than 3 (Lorange et al. 2005). Second, the number of *Y. pestis* bacteria transmitted by blocked fleas is several order of magnitudes higher (Lorange et al. 2005). Given that regurgitation of a larger inoculum increases the chance of the bacteria to establish a successful infection in the mammalian host, the ratio $\frac{\beta_{B}}{\beta_{V}}$ is likely to be higher than a few hundreds. Obviously, obtaining more accurate estimates of transmission and mortality rates in *X. cheopsis* (but also in other flea species) is particularly important to conclude on the adaptive nature of blockage.

Our analysis also introduces the possibility of within‐flea competition between different variants of *Y. pestis*. In particular, we contend that the production of a biofilm may be a way to outcompete other bacteria in the foregut of the flea. Within‐flea competition adds another dimension in the adaptive value of blockage. In particular, conditions (5) and (6) indicate that this mechanism is likely to promote the evolution of blockage. Recent experimental studies have explored the outcome of competition between different strains of *Y. pestis* in fleas (Rempe et al. 2012; Spinner et al. 2013; Vadyvaloo and Hinz 2015; Fukuto et al. 2018). These studies revealed that fitness costs are associated with mutations in several genes involved in flea‐borne transmission (*hfq* (Rempe et al. 2012), *rovM* (Vadyvaloo and Hinz 2015), and *phoP* (Fukuto et al. 2018)). Unfortunately, experiments following the competition taking place between *hms* variants in the flea remain to be carried out.

Empirical evidence of plague dynamics reveal the highly epidemic nature of plague outbreaks that is likely to be driven by seasonal variations of the environment (Stenseth et al. 2008; Moore et al. 2015; Schmid et al. 2015; Ngeleja et al. 2018). In such a fluctuating environment, our analysis reveals that selection for blockage is likely to vary through time. Blockage should be more strongly selected at the onset of epidemics, when many hosts are uninfected. In contrast, blockage is expected to decrease when the epidemic is fading away because a smaller number of susceptible hosts are available. This transient selection for higher rates of transmission (in spite on the induced mortality of the flea) is in line with previous studies that showed how the evolution of life‐history traits of pathogens can be altered by epidemiological dynamics (Day and Gandon 2007; Mideo et al. 2011; Berngruber et al. 2013). It would be interesting to study the variability of the ability to produce a biofilm and to block the fleas in natural populations. Analysis of bacteria sampled at different points in space or in time would allow to test our prediction that temporal fluctuations in the environment drives the maintenance of variability in *Y. pestis* populations.

Although our model tries to capture multiple routes of transmission, it is important to acknowledge that plague transmission involves a multitude of host species (Yang and Anisimov 2016). Our model, however, focuses on a simple scenario with a single species of vertebrate host and a single species of flea. Yet, the competence of fleas, their propensity to develop blockage, and their mortality rates (after blockage) are known to differ widely (Bland and Hinnebusch 2016; Hinnebusch et al. 2017; Hinnebusch et al. 2017). Besides, the infectious blood source is also known to affect the development of *Y. pestis* in the fleas (Bland et al. 2018). A full understanding of the ecology and evolution of the plague thus requires a more comprehensive description of the network of host and vector species involved in its transmission.

## Materials and Methods

### DERIVATION OF ***R***_0_

The ability of the pathogen to invade an uninfected host population is determined by, *R*_0_, its basic reproduction ratio. To derive *R*_0_, we need to consider the dynamics of equation (1) at the disease free equilibrium when $N_{H}=\frac{\lambda_{H}}{m_{H}}$ and $N_{F}=\frac{\lambda_{F}}{m_{F}}$:

$$\dot{X}=\left(F-M\right).X,$$

where

$$X=\left(\begin{array}{c} \begin{array}{c} I\\ F_{U} \end{array}\\ \begin{array}{c} F_{B}\\ P \end{array} \end{array}\right),$$

$$F=N_{H}\left(\begin{array}{cccc} \beta_{H} & \;\sigma \beta_{U} & \;\sigma \beta_{B} & \;\beta_{P}\\ 0 & \;0 & \;0 & \;0\\ 0 & \;0 & \;0 & \;0\\ 0 & \;0 & \;0 & \;0\; \end{array}\right),$$

$$M=\left(\begin{array}{cccc} m_{H}+\alpha_{H} & \;0 & \;0 & \;0\\ -\sigma N_{F} & \;m_{F}+\alpha_{U}+\varepsilon & \;-\gamma & \;0\\ 0 & \;-\varepsilon & \;m_{F}+\alpha_{B}+\gamma & \;0\\ -\theta & \;0 & \;0 & \;\delta \end{array}\right).$$

The basic reproduction ratio is the dominant eigenvalue of ***F***.***M*** ^−1^, which yields equation (3) in the main text.

### PATHOGEN EVOLUTION

To study pathogen evolution, we first track the dynamics of a rare mutant invading the population of a resident pathogen when the system has reached an endemic equilibrium. For the sake of simplicity, we assume that coinfections with the resident and the mutant pathogens are not feasible but we do allow for superinfections in the vector, which yields the dynamical system (1). In matrix form this yields the following dynamical system:

$${\dot{X}}_{m}=\left(F_{m}-M_{m}\right).X_{m},$$

where

$$X_{m}=\left(\begin{array}{c} \begin{array}{c} I_{m}\\ F_{U,m} \end{array}\\ \begin{array}{c} F_{B,m}\\ P_{m} \end{array} \end{array}\right),$$

$$F_{m}=\bar{S}\left(\begin{array}{cccc} \beta_{H} & \;\sigma \beta_{U} & \;\sigma \beta_{B} & \;\beta_{P}\\ 0 & \;0 & \;0 & \;0\\ 0 & \;0 & \;0 & \;0\\ 0 & \;0 & \;0 & \;0\; \end{array}\right),$$

$$M_{m}=\left(\begin{array}{cccc} m_{H}+\alpha_{H} & \;0 & \;0 & \;0\\ -\sigma {\bar{F}}_{S}-\sigma S_{1} & \;m_{F}+\alpha_{U}+\varepsilon_{m}+\sigma S_{2} & \;-\gamma & \;0\\ 0 & \;-\varepsilon_{m} & \;m_{F}+\alpha_{B}+\gamma & \;0\\ -\theta & \;0 & \;0 & \;\delta \end{array}\right)$$

with $S_{1}=s\left[\varepsilon ,\varepsilon_{m}\right]{\bar{F}}_{U}$ and $S_{2}=\;s\left[\varepsilon_{m},\varepsilon \right]\bar{I}$ .

The basic reproduction ratio is the dominant eigenvalue of $F_{m}.{M_{m}}^{-1}$, which yields equation (4) in the main text.

### SIMULATIONS

In Figure 3, we present a simulation of the dynamical system (1) with two strains and no superinfection: one strain never blocks the flea ($\varepsilon_{1}=0$) and another strain can block infected fleas ($\varepsilon_{2}=1$). To illustrate the dynamics occurring during an epidemic, we assumed that none of the vectors are initially infected ($F_{S}\left(0\right)=\frac{\lambda_{F}}{m_{F}}$) and we introduced a small density of infected hosts: $I_{1}\left(0\right)=10^{-3}$, $I_{2}\left(0\right)=10^{-4}$, and $S\left(0\right)=\frac{\lambda_{H}}{m_{H}}$. Figure 3 shows the epidemiological and the evolutionary dynamics when condition (5) is satisfied or not (panel (B) and (A), respectively).

In Figure 4, we present a simulation of the dynamical system (1) under the assumption that $\lambda_{F}\left(t\right)=1+Sin\left(2\pi t/T\right)$ varies periodically because of seasonality (T=200). We assume that two bacterial strains are competing but there is no superinfection: one strain never blocks the flea ($\varepsilon_{1}=0$) and another strain can block infected fleas ($\varepsilon_{2}=1$). Under the parameter values we chose, the two strains can coexist in the long term, although the second strain (the strain producing a biofilm) should be outcompeted by the first strain in a constant environment. We show the epidemiological and evolutionary dynamics for three consecutive seasons, when the system has reached a stable limit cycle. We obtained qualitatively similar results in a modified model in which the growth rates of the two hosts are density dependent (not shown). The evolutionary dynamics does depend on the densities in the different compartments but the threshold quantities we identify on our analysis, equations (5), (6) and (9) are robust to such modifications of the regulation of population densities.

Associate Editor: A. Gardner

## Supporting information


**Figure S1**: The superinfection function.

**Table S1**: Definitions of the main parameters of the model.

## References

[evl3111-bib-0002] Achtman, M. , K. Zurth , G. Morelli , G. Torrea , A. Guiyoule , et al. 1999 *Yersinia pestis*, the cause of plague, is a recently emerged clone of *Yersinia pseudotuberculosis* . Proc. Natl. Acad. Sci. USA 96:14043–14048. 10570195 10.1073/pnas.96.24.14043 PMC24187

[evl3111-bib-0012] Bacot, A. W. , and C. J. Martin . 1914 Observations on the mechanism of the transmission of plague by fleas. J Hyg (Lond) 13(Suppl):423–439. 20474555 PMC2167459

[evl3111-bib-0030] Berngruber, T. W. , R. Froissart , M. Choisy , and S. Gandon . 2013 Evolution of virulence in emerging epidemics. PLoS Pathog. 9:e1003209. 23516359 10.1371/journal.ppat.1003209 PMC3597519

[evl3111-bib-0042] Bland, D. M. , C. O. Jarrett , C. F. Bosio , and B. J. Hinnebusch . 2018 Infectious blood source alters early foregut infection and regurgitative transmission of *Yersinia pestis* by rodent fleas. PLoS Pathog. 14:e1006859. 29357385 10.1371/journal.ppat.1006859 PMC5794196

[evl3111-bib-0041] Bland, D. M. , and B. J. Hinnebusch . 2016 Feeding behavior modulates biofilm‐mediated transmission of *Yersinia pestis* by the cat flea, *Ctenocephalides felis* . PLoS Negl. Trop. Dis. 10:e0004413. 26829486 10.1371/journal.pntd.0004413 PMC4734780

[evl3111-bib-0001] Bramanti, B. , N. C. Stenseth , L. Walløe , and X. Lei . 2016 Plague: a disease which changed the path of human civilization. Adv. Exp. Med. Biol. 918:1–26. 27722858 10.1007/978-94-024-0890-4_1

[evl3111-bib-0006] Chouikha, I. , and B. J. Hinnebusch . 2012 Yersinia–flea interactions and the evolution of the arthropod‐borne transmission route of plague. Curr. Opin. Microbiol. 15:239–246. 22406208 10.1016/j.mib.2012.02.003 PMC3386424

[evl3111-bib-0010] Chouikha, I. , and B. J. Hinnebusch . 2014 Silencing urease: a key evolutionary step that facilitated the adaptation of *Yersinia pestis* to the flea‐borne transmission route. PNAS 111(52):18709–18714. 25453069 10.1073/pnas.1413209111 PMC4284590

[evl3111-bib-0028] Day, T. , and S. Gandon . 2006 Insights from Price's equation into evolutionary. In: Disease evolution: models, concepts, and data analyses, Vol. 71, [Feng Z., Dieckmann U., and Levin S., eds.]. American Mathematical Society, Providence, Rhode Island, p. 23.

[evl3111-bib-0029] Day, T. , and S. Gandon 2007 Applying population‐genetic models in theoretical evolutionary epidemiology. Ecol. Lett. 10:876–888. 17845288 10.1111/j.1461-0248.2007.01091.x

[evl3111-bib-0015] Eisen, R. J. , A. P. Wilder , S. W. Bearden , J. A. Montenieri , and K. L. Gage . 2007 Early‐phase transmission of *Yersinia pestis* by unblocked Xenopsylla cheopis (Siphonaptera: Pulicidae) is as efficient as transmission by blocked fleas. J. Med. Entomol. 44(4):678–682. 17695025 10.1603/0022-2585(2007)44[678:etoypb]2.0.co;2

[evl3111-bib-0017] Eisen, R. J. , D. T. Dennis , and K. L. Gage . 2015 The role of early‐phase transmission in the spread of *Yersinia pestis* . J. Med. Entomol. 52:1183–1192. 26336267 10.1093/jme/tjv128 PMC4636957

[evl3111-bib-0016] Eisen, R. J. , J. M. Petersen , C. L. Higgins , D. Wong , C. E. Levy , P. S. Mead , et al. 2008 Persistence of *Yersinia pestis* in soil under natural conditions. Emerg. Infect. Dis. 14:941–943. 18507908 10.3201/eid1406.080029 PMC2600287

[evl3111-bib-0014] Eisen, R. J. , S. W. Bearden , A. P. Wilder , J. A. Montenieri , M. F. Antolin , and K. L. Gage . 2006 Early‐phase transmission of *Yersinia pestis* by unblocked fleas as a mechanism explaining rapidly spreading plague epizootics. Proc. Natl. Acad. Sci. USA 103:15380–15385. 17032761 10.1073/pnas.0606831103 PMC1592641

[evl3111-bib-0038] Fukuto, H. S. , V. Vadyvaloo , J. B. McPhee , H. N. Poinar , E. C. Holmes , and J. B. Bliska . 2018 A single amino acid change in the response regulator PhoP, acquired during *Yersinia pestis* evolution, affects PhoP target gene transcription and Polymyxin B susceptibility. J. Bacteriol. 200:e00050‐18. 29440252 10.1128/JB.00050-18 PMC5892123

[evl3111-bib-0043] Gage, K. L. , and M. Y. Kosoy . 2005 Natural history of plague: perspectives from more than a century of research. Annu. Rev. Entomol. 50:505–528. 15471529 10.1146/annurev.ento.50.071803.130337

[evl3111-bib-0044] Gage, K. , and M. Kosoy . 2006 Recent trends in plague ecology In: Recovery of the black‐footed ferret: progress and continuing challenges [Roelle J., Miller B., Godbey J., and Biggins D., eds.]. U.S. Geological Survey, Fort Collins, Colarado, pp. 213–232.

[evl3111-bib-0022] Geritz, SaH. , E. Kisdi , G. Mesze´NA , and JaJ. Metz . 1998 Evolutionarily singular strategies and the adaptive growth and branching of the evolutionary tree. Evol. Ecol. 12:35–57.

[evl3111-bib-0008] Hinnebusch, B. J. , A. E. Rudolph , P. Cherepanov , J. E. Dixon , T. G. Schwan , and Å. Forsberg . 2002 Role of *Yersinia* murine toxin in survival of *Yersinia pestis* in the midgut of the flea vector. Science 296:733–735. 11976454 10.1126/science.1069972

[evl3111-bib-0007] Hinnebusch, B. J. , C. O. Jarrett , and D. M. Bland . 2017 ‘Fleaing’ the plague: adaptations of *Yersinia pestis* to its insect vector that lead to transmission. Annu. Rev. Microbiol. 71:215–232. 28886687 10.1146/annurev-micro-090816-093521

[evl3111-bib-0034] Hinnebusch, B. J. , D. M. Bland , C. F. Bosio , and C. O. Jarrett . 2017 Comparative ability of oropsylla montana and xenopsylla cheopis fleas to transmit *Yersinia pestis* by two different mechanisms. PLoS Negl. Trop. Dis. 11:e0005276. 28081130 10.1371/journal.pntd.0005276 PMC5230758

[evl3111-bib-0004] Hinnebusch, B. J. , I. Chouikha , and Y.‐C. Sun . 2016 Ecological opportunity, evolution, and the emergence of flea‐borne plague. Infect. Immun. 84:1932–1940. 27160296 10.1128/IAI.00188-16 PMC4936347

[evl3111-bib-0011] Hinnebusch, B. J. , R. D. Perry , and T. G. Schwan . 1996 Role of the *Yersinia pestis* hemin storage (hms) locus in the transmission of plague by fleas. Science 273(5273):367–370. 8662526 10.1126/science.273.5273.367

[evl3111-bib-0025] Hurford, A. , D. Cownden , and T. Day . 2010 Next‐generation tools for evolutionary invasion analyses. J. R. Soc. Interface 7:561–571. 19955121 10.1098/rsif.2009.0448 PMC2842787

[evl3111-bib-0023] Kisdi, É. , and S. A. H. Geritz . 2010 Adaptive dynamics: a framework to model evolution in the ecological theatre. J. Math. Biol. 61:165–169. 19777234 10.1007/s00285-009-0300-9

[evl3111-bib-0031] Lélu, M. , M. Langlais , M.‐L. Poulle , E. Gilot‐Fromont , and S. Gandon . 2013 When should a trophically and vertically transmitted parasite manipulate its intermediate host? The case of *Toxoplasma gondii* . Proc. Biol. Sci. 280:20131143. 23825211 10.1098/rspb.2013.1143 PMC3712452

[evl3111-bib-0019] Lorange, E. A. , B. L. Race , F. Sebbane , and B. J. Hinnebusch . 2005 Poor vector competence of fleas and the evolution of hypervirulence in *Yersinia pestis* . J. Infect. Dis. 191:1907–1912. 15871125 10.1086/429931

[evl3111-bib-0021] Metz, J. A. , R. M. Nisbet , and S. A. Geritz . 1992 How should we define ‘fitness’ for general ecological scenarios? Trends Ecol. Evol. 7:198–202. 21236007 10.1016/0169-5347(92)90073-K

[evl3111-bib-0039] Mideo, N. , W. A. Nelson , S. E. Reece , A. S. Bell , A. F. Read , and T. Day . 2011 Bridging scales in the evolution of infectious disease life histories: application. Evolution 65:3298–3310. 22023593 10.1111/j.1558-5646.2011.01382.x PMC3937741

[evl3111-bib-0013] Mollaret, H. H. 1963 Conservation expérimentale de la peste dans le sol. Bull. Soc. Pathol. Exotique 6:1168–1182. 14156818

[evl3111-bib-0032] Moore, S. M. , A. Monaghan , J. N. Borchert , J. T. Mpanga , L. A. Atiku , K. A. Boegler , et al. 2015 Seasonal fluctuations of small mammal and flea communities in a Ugandan plague focus: evidence to implicate *Arvicanthis niloticus* and *Crocidura* spp. as key hosts in *Yersinia pestis* transmission. Parasit Vectors 8:11. 25573253 10.1186/s13071-014-0616-1 PMC4297414

[evl3111-bib-0033] Ngeleja, R. C. , L. S. Luboobi , and Y. Nkansah‐Gyekye . 2018 Plague disease model with weather seasonality. Math. Biosci. 302:80–99. 29800562 10.1016/j.mbs.2018.05.013

[evl3111-bib-0035] Rempe, K. A. , A. K. Hinz , and V. Vadyvaloo . 2012 Hfq regulates biofilm gut blockage that facilitates flea‐borne transmission of *Yersinia pestis* . J. Bacteriol. 194:2036–2040. 22328669 10.1128/JB.06568-11 PMC3318476

[evl3111-bib-0027] Schmid, B. V. , U. Büntgen , W. R. Easterday , C. Ginzler , L. Walløe , B. Bramanti , et al. 2015 Climate‐driven introduction of the Black Death and successive plague reintroductions into Europe. Proc. Natl. Acad. Sci. USA 112:3020–3025. 25713390 10.1073/pnas.1412887112 PMC4364181

[evl3111-bib-0009] Sebbane, F. , A. Devalckenaere , J. Foulon , E. Carniel , and M. Simonet . 2001 Silencing and reactivation of urease in *Yersinia pestis* is determined by one G residue at a specific position in the ureD gene. Infect. Immun. 69(1):170–176. 11119503 10.1128/IAI.69.1.170-176.2001 PMC97869

[evl3111-bib-0020] Sebbane, F. , C. Jarrett , D. Gardner , D. Long , and B. J. Hinnebusch . 2009 The *Yersinia pestis* caf1M1A1 fimbrial capsule operon promotes transmission by flea bite in a mouse model of bubonic plague. Infect. Immun. 77:1222–1229. 19103769 10.1128/IAI.00950-08 PMC2643634

[evl3111-bib-0036] Spinner, J. L. , A. B. Carmody , C. O. Jarrett , and B. J. Hinnebusch . 2013 Role of *Yersinia pestis* toxin complex (Tc) family proteins in resistance to phagocytosis by polymorphonuclear leukocytes. Infect. Immun. 81:4041–4052. 23959716 10.1128/IAI.00648-13 PMC3811843

[evl3111-bib-0026] Stenseth, N. C. , B. B. Atshabar , M. Begon , S. R. Belmain , E. Bertherat , E. Carniel , et al. 2008 Plague: past, present, and future. PLoS Med. 5:e3. 10.1371/journal.pmed.0050003 PMC2194748 18198939

[evl3111-bib-0045] Sun Y. C. , B. J. Hinnebusch , C. Darby . 2008 Experimental evidence for negative selection in the evolution of a Yersinia pestis pseudogene. Proc Natl Acad Sci USA. 105:8097–101. 18523005 10.1073/pnas.0803525105 PMC2430365

[evl3111-bib-0003] Sun, Y.‐C. , C. O. Jarrett , C. F. Bosio , and B. J. Hinnebusch . 2014 Retracing the evolutionary path that led to flea‐borne transmission of *Yersinia pestis* . Cell Host Microbe 15:578–586. 24832452 10.1016/j.chom.2014.04.003 PMC4084870

[evl3111-bib-0037] Vadyvaloo, V. , and A. K. Hinz . 2015 A LysR‐Type transcriptional regulator, RovM, senses nutritional cues suggesting that it is involved in metabolic adaptation of *Yersinia pestis* to the flea gut. PLoS One 10:e0137508. 26348850 10.1371/journal.pone.0137508 PMC4562620

[evl3111-bib-0005] Valtueña, A. A. , A. Mittnik , F. M. Key , W. Haak , R. Allmäe , A. Belinskij , et al. 2017 The stone age plague and its persistence in Eurasia. Curr. Biol. 27:3683–3691.e8. 29174893 10.1016/j.cub.2017.10.025

[evl3111-bib-0024] Waxman, D. , and S. Gavrilets . 2005 20 questions on adaptive dynamics. J. Evol. Biol. 18:1139–1154. 16135102 10.1111/j.1420-9101.2005.00948.x

[evl3111-bib-0018] Webb, C. T. , C. P. Brooks , K. L. Gage , and M. F. Antolin . 2006 Classic flea‐borne transmission does not drive plague epizootics in prairie dogs. Proc. Natl. Acad. Sci. USA 103:6236–6241. 16603630 10.1073/pnas.0510090103 PMC1434514

[evl3111-bib-0040] Yang R., and Anisimov A., Eds. 2016 Yersinia pestis: retrospective and perspective. Springer, the Netherlands.

