# Supplementary figures and images for: To block or not to block: The adaptive manipulation of plague transmission
Source: Evol Lett. 2019 Mar 27;3(2):152–61. doi: 10.1002/evl3.111 (PMC6541909; doi:10.1002/evl3.111)

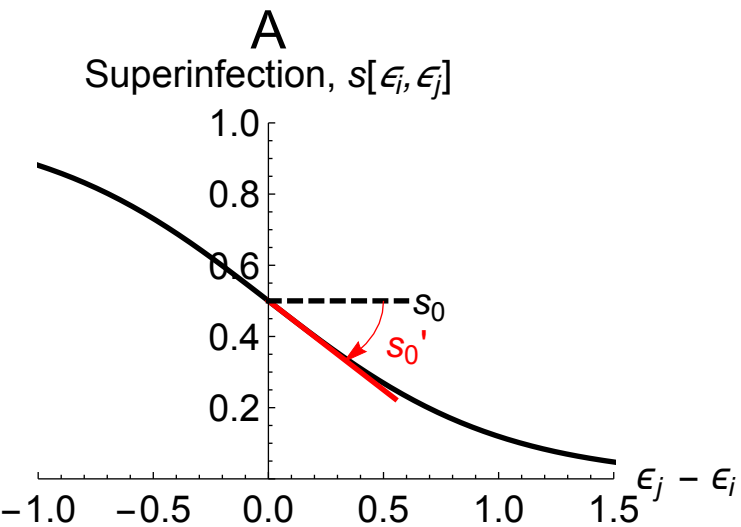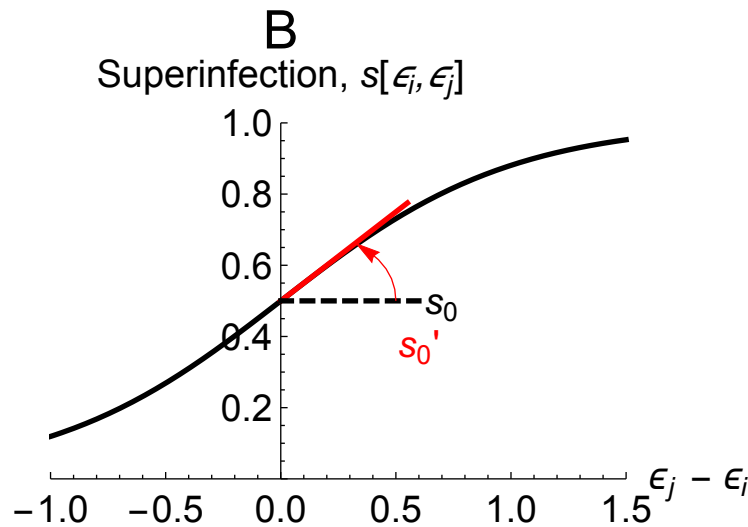

Supplement: Supplementary file 1 — Figure S1: The superinfection function. [file EVL3-3-152-s001.pdf]
