# Supplementary material for: To block or not to block: The adaptive manipulation of plague transmission
Source: Evol Lett. 2019 Mar 27;3(2):152–61. doi: 10.1002/evl3.111 (PMC6541909; doi:10.1002/evl3.111)
Supplement: Supplementary file 2 — Table S1: Definitions of the main parameters of the model. [file EVL3-3-152-s002.docx]

**Table S1: Definitions of the main parameters of the model**

| **Main parameters** | **Definitions** |
| --- | --- |
| $\lambda_{H}$ | Intrinsic growth rate of the vertebrate population |
| $N_{H}=S+I$ | Vertebrate host density: susceptible + infected |
| $\lambda_{F}$ | Intrinsic growth rate of the flea population |
| $N_{F}=F_{S}+F_{U}+F_{B}$ | Flea density: susceptible + infected-unblocked + infected-blocked |
| $P$ | Density of free-living *Y. pestis* bacteria in the soil |
| $\sigma$ | Feeding rate of fleas |
| $s_{0}$, ${s'}_{0}$ | Parameters governing the shape of the superinfection function |
| $\beta_{P}$ | Infectivity of free-ling propagules for the vertebrate host |
| $\beta_{H}$ | Direct transmission rate among vertebrate hosts |
| $\beta_{B}$, $\beta_{U}$ | Infectivity of blocked and unblocked fleas |
| $m_{H}$ | Natural mortality rate of the host |
| $m_{F}$ | Natural mortality rate of the flea |
| $\delta$ | Mortality rate of free-living propagules |
| $\theta$ | Rate of production of free-living propagules by infected hosts |
| $\alpha_{H}$ | Virulence (mortality induced by the pathogen) in the host |
| $\alpha_{U}$ | Virulence (mortality induced by the pathogen) in the unblocked fleas |
| $\alpha_{B}$ | Virulence (mortality induced by the pathogen) in the blocked fleas |
